# Applying the Non-adoption, Abandonment, Scale-up, Spread and Sustainability (NASSS) framework to evaluate automated evidence synthesis in health behaviour change

**DOI:** 10.1177/13591053241229870

**Published:** 2024-03-08

**Authors:** Peter Branney, Marta M Marques, Emma Norris

**Affiliations:** 1 University of Bradford, UK; 2 Universidade Nova de Lisboa, Portugal; 3 Brunel University London, UK

**Keywords:** automated evidence synthesis, health behaviour change, Human Behaviour-Change Project, NASSS framework

## Abstract

Automated tools to speed up the process of evidence synthesis are increasingly apparent within health behaviour research. This brief review explores the potential of the Non-adoption, Abandonment, Scale-up, Spread and Sustainability framework for supporting automated evidence synthesis in health behaviour change by applying it to the ongoing Human Behaviour-Change Project, which aims to revolutionize evidence synthesis within behaviour change intervention research. To increase the relevance of NASSS for health behaviour change, we recommend i) terminology changes (‘condition’ to ‘behaviour’ and ‘patient’ to ‘end user’) and ii) that it is used prospectively address complexities iteratively. We draw conclusions about i) the need to specify the organizations that will use the technology, ii) identifying what to do if interdependencies fail and iii) even though we have focused on automated evidence synthesis, NASSS would arguably be beneficial for technology developments in health behaviour change more generally, particularly for invention development.

The aim of this brief review is to outline the potential of the Non-adoption, Abandonment, Scale-up, Spread and Sustainability framework (NASSS; Greenhalgh et al., 2017) to support and evaluate the development of automated evidence synthesis tools in health behaviour change. Evidence synthesis methodologies, such as systematic reviews and meta-analyses, are essential to interpret complex bodies of knowledge in any given domain, such as health behaviour change (Michie et al., 2017). However, evidence synthesis outputs are often written for an academic audience and hence may be of limited use in commissioning, implementing and evaluating health services (Glasziou et al., 2014). Additionally, evidence synthesis research is highly resource-intensive, with reviews often out-of-date by the time of completion (Elliott et al., 2014). Automated tools are being applied to speed up the process of evidence synthesis in the behavioural and health sciences (Marshall et al., 2020), such as automated data extraction (Jonnalagadda et al., 2015) and crowd-sourcing of article screening (Noel-Storr et al., 2021). Living evidence reviews typically use automated evidence synthesis (Millard et al., 2019; Thomas et al., 2017), to support the process of updating the review as new papers become available (e.g., Cochrane Collaboration, 2019; Simons et al., 2021)

## The Human Behaviour-Change Project

The Human Behaviour-Change Project (HBCP) applies artificial intelligence to automatically synthesize evidence on behaviour change interventions from published intervention reports (Mac Aonghusa and Michie, 2021), to provide solutions to the ‘big question’ of behaviour change: ‘What works, compared to what, for what behaviours, how well, for how long, with whom, in what setting and why?’ (Michie et al., 2020b). Data from intervention evaluation reports of randomized controlled trials is extracted into the HBCP knowledge system using the structure of a Behaviour Change Intervention Ontology (BCIO; Michie et al., 2020b). The BCIO, as consistent with other ontologies, provides a set of (1) unique, unambiguous entities (such as objects, attributes and processes), (2) labels and definitions for these entities and (3) specified relationships between these entities (Arp et al., 2015), within the specific context of behaviour change interventions.

The HBCP knowledge system has to-date been piloted using smoking cessation behaviour change intervention papers included within Cochrane reviews (Bonin et al., 2020) and physical activity intervention papers (Michie et al., 2020b). HBCP aims to identify where the most effective interventions and robust evidence exists (‘known knowns’) and gaps in research to be filled (‘known unknowns’; Hagger et al., 2020). When launched, the online HBCP web-system aims to have interfaces tailored to different stakeholders, e.g public, practitioners and policy-makers (Michie et al., 2017). In relation to Technology Readiness Levels (EARTO, 2014), HBCP can be seen as currently at Level 3 (proof of concept) or 4 (validation of prototype in laboratory), with user testing underway in researcher, practitioner and public groups.

## The Non-adoption, Abandonment, Scale-up, Spread and Sustainability (NASSS) framework

NASSS is a framework for understanding the non-adoption, abandonment, scale-up, spread and sustainability of technology within a complex healthcare system composed of many interacting entities (Abimbola et al., 2019; Greenhalgh et al., 2018). The NASSS framework has seven domains, each with a set of questions to evaluate the health technology (see Table 1). According to each domain, a system is evaluated either retrospectively or prospectively as ‘simple’ (straightforward, predictable, and with few components), ‘complicated’ (multiple interacting components or issues) or ‘complex’ (dynamic, not easily disaggregated into constituent components, and unpredictable; Greenhalgh et al., 2017). Example technologies evaluated using NASSS include electronic decision support in cardiovascular treatment (Abimbola et al., 2019) and internet-delivered CBT for insomnia (Kadesjö Banck and Bernhardsson, 2020). Our application of the NASSS framework to HBCP in this brief review is based on the available published evidence on the HBCP via papers, information on the project’s Open Science Framework pages (West et al., 2016) and the project’ website.^1^

**Table 1.** Application of the Non-adoption, Abandonment, Scale-up, Spread and Sustainability (NASSS) framework to the Human Behaviour-Change Project (HBCP).

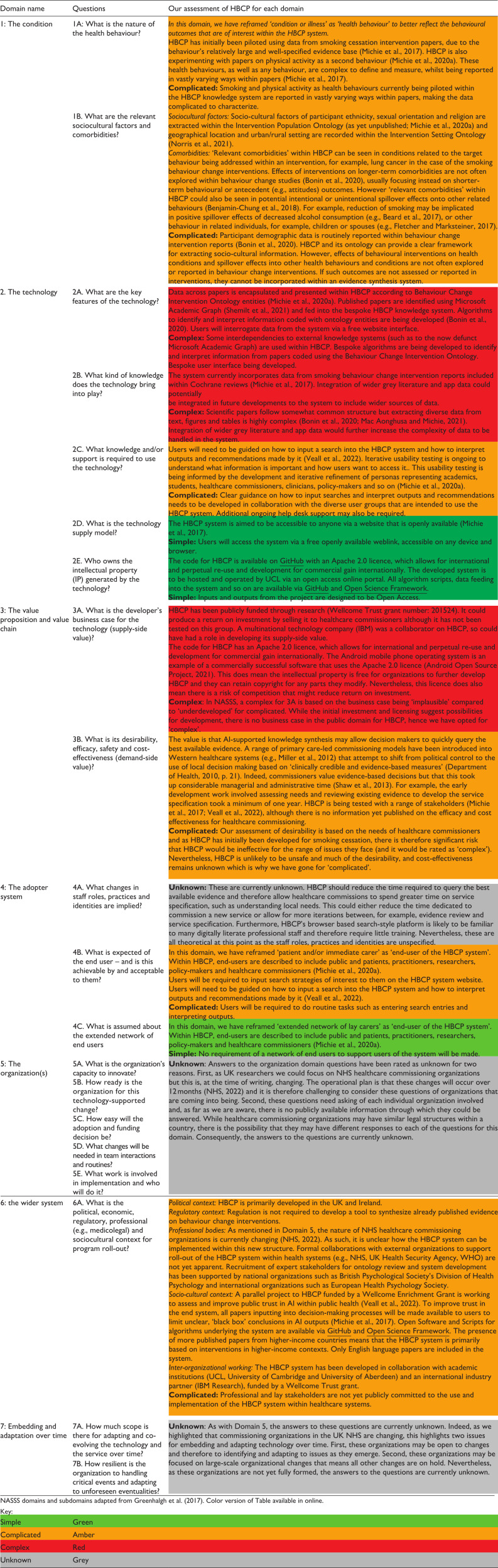

## Using NASSS to inform automated evidence synthesis projects in health behaviour change

Our application of NASSS to HBCP is illustrated in Table 2. From this application, we propose two recommendations to increase the relevance of NASSS to health behaviour change. First, terminology used within NASSS could be adjusted to better relate to health behaviour change contexts. In Domains 1, changing the ‘condition’ to ‘behaviour’ would allow clearer specification of outcome behaviours within interventions, and also reflect that behaviour’s influence multiple conditions and/or illnesses. HBCP, for example, has been initially piloted on smoking cessation interventions, not for a specific health condition. In Domain 4, changing ‘patient’ to ‘end user’ would better reflect that end users of behaviour change interventions are not necessarily patients. These adjustments would allow NASSS to consider the benefit of health technologies for behaviour change and avoid a narrow focus on ill health.

**Table 2.** Illustration of NASSS domain 1, questions and their use.*

Question	Simple	Complicated	Complex
1A. What is the nature of the condition or illness?	Well-characterized, well-understood, predictable	Not fully characterized, understood of predictable	Poorly characterized, poorly understood, unpredictable or high risk
Examples	Follow-up consultations after cancer surgery and routine consultations for young adults with diabetes were fairly predictable and consistent; the few unpredictable eventualities were low-risk. Diabetes in pregnancy is a volatile condition and if poorly controlled can lead to foetal malformations or death. The physician felt strongly accountable to the unborn child so ‘erred on the side of caution’, inviting very few women to try the service. Heart failure was typically an unpredictable condition whose effects varied from patient to patient (and in the same patient over time).		
1B. What are the relevant sociocultural factors and comorbidities?	Unlikely to affect care significantly	Must be factored into care plan and service model	Pose significant challenges to care planning and service provision.
Examples	In the heart failure service, nurses made judgements about patients’ cognitive ability, health literacy, motivation and mental health status (notably depression), leading to many not being offered the video consultation option. In the antenatal diabetes service, most patients who were invited to try video consultations (3% of the clinic population) were native English speakers, university educated and working in professional jobs (e.g. doctor, science teacher); most patients in this clinic were first or second generation immigrants (many with limited English) and school education only. Unless the patient had very high health literacy, IT literacy and English fluency, they was not offered the option of video consultations.		

* This table merges table 2 and Multimedia appendix 2 from Greenhalgh et al. (2017).

Second, we recommend that a full, prospective NASSS evaluation be performed at the early stages of project conceptualization, such as at grant writing stage, to ensure implementation complexities are identified and addressed iteratively from the project’s start. Although the NASSS was developed for either prospective or retrospective application (Greenhalgh et al., 2017) and it is beneficial to retrospectively consider what has led to the success or failure of different technologies, we argue that the utility of NASSS assessment is in prospectively considering potential challenges in technology development to ensure scale-up, spread and sustainability. In other related applications of NASSS, Shaw et al. (2013) used it as a framework for exploring machine learning in healthcare generally but our suggestion is that it should be used on specific technologies, such as HBCP. Meinert et al. (2020) suggested iterative use of NASSS for an app to reduce social isolation during COVID-19 social distancing measures, similar to our suggestion but providing little detail. Similar to the RAG rating system in project management, where a technology is assessed as either complicated or complex, we would need to find ways of making them simpler. Where this is impractical for some domains, it is nevertheless important for the technology’s success to do so across as many domains as possible.

We applied the NASSS to HBCP as a case study (Table 2) and drew three main conclusions. First, specifying the organizations (Domain 5) that would use the technology would have minimized the questions we could not answer (the ‘unknowns’). Second, as essential interdependencies with other systems make evidence synthesis technologies complex, it is important to plan what will happen if interdependencies fail. Last, HBCP is an ‘ambitious project’ (Michie et al., 2017: 11) and we would therefore not expect all or most NASSS domains to be assessed as ‘simple’ but using NASSS is nevertheless an important exercise to help us think about the uptake of automated evidence synthesis.

## Conclusion

In this brief review, we have argued that NASSS should be used prospectively to enhance the development of sustainable automated evidence synthesis technologies for health behaviour change. NASSS would also arguably be beneficial for technology developments across health behaviour change more generally, such as in intervention development. We hope that with the example provided in this brief review, other health behaviour change researchers and interventionists can use this as a basis to implement NASSS in their technology projects or products.

## Notes

1.
https://www.humanbehaviourchange.org/
